# Energy efficient gateway based routing with maximized node coverage in a UAV assisted wireless sensor network

**DOI:** 10.1371/journal.pone.0295615

**Published:** 2023-12-27

**Authors:** Bilal Ahmad, Masroor Ahmed, Nadeem Anjum, Masood Ur Rehman, Naeem Ramzan

**Affiliations:** 1 Capital University of Science & Technology, Islamabad, Pakistan; 2 James Watt School of Engineering, University of Glasgow, Glasgow, United Kingdom; 3 School of Computing Engineering and Physical Sciences, University of the West of Scotland, Paisley, United Kingdom; SUMAIT University, TANZANIA, UNITED REPUBLIC OF

## Abstract

Ad-hoc wireless sensor networks face challenges of optimized node deployment for maximizing coverage and efficiently routing data to control centers in post disaster events. These challenges impact the outcome for extending the lifetime of wireless sensor networks. This study presents a uav assisted reactive zone based EHGR (energy efficient hierarchical gateway routing protocol) that is deployed in a situation where the natural calamity has caused communication and infrastructure damage to a major portion of the sensor network. EHGR is a hybrid multi layer routing protocol for large heterogeneous sensor nodes (smart nodes, basic nodes, user handheld devices etc.) EHGR is tailored to meet two important concerns for a disaster hit wsn ie. optimized deployment and energy efficient routing. The first part of EGHR focuses on maximized coverage during node deployments. Maximized coverage is an important aspect to be considered during the event of disaster since most of the nodes loose coverage and are detached from the wireless sensor network. The first part of EHGR uses state of the art game theory approach to build a model that maximizes the coverage of nodes during the deployment phase from all participating entities i.e. nodes and uavs. Rather than fixing the cluster head as is the case in traditional cluster-based approaches EHGR uses the energy centroid nodes. Energy centroid nodes evolve on the basis of aggregated energy of the zone. This approach is superior to simply electing cluster head nodes on the basis of some probability function. The nodes that fail to achieve any successful outcome from the game theory matching model fail to get any association. These nodes will use multi hop d2d relay approach to reach the energy centroid nodes. Gateway relay nodes used with the game theory approach during the deployment of the uav assisted wsn improves the overall coverage by 25% against traditional leach based hierarchical approaches. Once the optimum deployment phase is completed the routing phase is initiated. Aggregated data is sent by the energy centroid nodes from the ECN nodes to the servicing micro controller enabled un manned aerial vehicles. The routing process places partial burden of zone formation and data transmission to the control center for each phase on the servicing uavs. Energy centroid nodes engage only in the data aggregation process and transmission of data to servicing uav. Servicing-uavs reduce energy dissipated of the entire zone which result in gradual decrease of energy for the zone thus increasing the network lifetime. Node deployment phase and the routing phase of EHGR utilize the computations provide by the mirco controller enabled unmanned aerial vehicles such that the computationally intensive calculations are offloaded to the servicing uav. Experiment results indicate an increase in the first dead node report, half dead node report, and last dead node report. Network lifetime is extended to approximately 1800 rounds which is an increase by ratio of 100% against the traditional leach approach and increase by 50% percent against the latest approaches as highlighted in the literature.

## Introduction

Wireless sensor networks are being deployed largely in almost all areas of scientific & industrial domains. Much of this is due to the massive availability of smart devices that are capable of being connected with each other to form a network or IoT based network pointed out by (Fotouhi et al, 2020) and (Abdul-Qawy et al., 2021) respectively respectively in [1, 2]. Smart Iot devices are deployed in the region of interest where they gather information and transmit it to the nearby control centers for further processing. However during a natural calamity much of the network lose communication with on another and the control center. In these situations the ad-hoc deployment of wireless sensor networks will be beneficial as indicated by (Temene et al, 2022) in [3]. Ad-hoc wsns are deployed to reduce the network recovery time while providing necessary short term communication services to the devices. Ad hoc wsn can extend the functionality in providing temporary connectivity services in the outage areas to user hand held devices. Such deployments have many potential benefits such as extended reach and robustness, flexibility, network efficiency, reduced cost etc. Latest developments of the user equipment and advancements in the broadband technologies allow the wsn networks to support a rich set of applications for establishing connectivity between sensor node and user applications for a myriad list of service such as map sharing, live video conversation that have potentials to even save human lives. Ad-hoc wireless sensor networks consist of one sink node called the base station (BS) and multiple user equipment called sensor nodes pointed out by (AlAli et al, 2022) in [4]. Sensor nodes communicate with the base station over wireless links in order to reach the control center placed in a nearby vicinity. The sensor nodes or the user equipment are energy constrained low power devices. A crucial design factor for wsn is to develop algorithms at the different layers of the protocol stack (such as network layer, data link layer) such that the protocols developed don’t deplete the limited battery power of the sensor nodes in the respective layers thereby increasing the network lifetime. In this study the user equipment are the sensor nodes that are resource constraint. The sensor nodes are heterogeneous that can be used for different purposes to perform a variety of tasks such as surveillance, temperature monitoring, measuring soil moisture etc. The sensor nodes can be used in patient monitoring in a IoT based remote health care system. The sensor nodes thus can serve many different tasks and applications and therefore there capabilities can be enhanced by embedding a variety of technologies having different data transmission rate, frequency, distance coverage and power consumption such as zigbee, bluetooth, wi-max etc supported through by ad-hoc deployment of wireless sensor networks. Khan et al. pointed that once the data is gathered sensor nodes transmit the information to cluster heads or control centers placed in a nearby vicinity or through a fog based gateway in [5]. In heterogeneity some of the nodes can be advance nodes. Advance nodes are classified to have higher computational power, greater storage capacity and are capable to send data over a large distance as compared to normal sensor nodes. In the literature the researchers assume half of the nodes to be normal and half to be advance that participate in the wireless sensor networks. (Abdul-Qawy et al, 2022) indicated that the advanced nodes can be used as relay nodes in [2] however in this study entire zone establishment will be through the game theory approach and every node that satisfies the optimization function presented in the section labeled *energy efficient hierarchical gateway routing* can qualify to act as the energy centroid node or the relay node.

Recent trends in wireless sensor networks focusing on ad-hoc deployments in various regions use unmanned aerial vehicles (uavs) or aerial drones as pointed out by (Nguyen et al, 2021) in [6]. The uavs provide connectivity services to the sensor nodes by hovering over the deployed zones. Deployment of un-manned aerial vehicles in ad-hoc networks can be tailored to meet a particular on demand scenario or a disastrous scenario as is the case in this study where the un manned aerial vehicles can be used through a chip mounted micro controllers to route data to the control centers. The zones established in such scenarios are connected with a particular drones all considered a cell. After a careful analysis of the entire deployment zones it is also possible to identify total number of aerial drones that will be required to service the outage area. (Fotouhi et al, 2019) in [7] pointed out the ability of the drones to hover and move in the coverage area that can increase the distance coverage to optimize the required throughput level. Research on drones to be used as ad-hoc deployments for wsns is still in early stages. In ad-hoc uav assisted wireless sensor networks routing is a crucial point that has been mostly researched recently. Traditional routing algorithms such as the Bellmanford cannot be deployed for these networks due to heavy calculation involved in determining the shortest path towards the control centers. These calculations if used can result in early energy depletion thus reducing the lifetime of the sensor nodes and the active lifetime of the network. Many routing protocols have been presented by researchers focusing on energy perseverance issues a good example of these is presented by (Khan et al, 2021) in [5] and by (Sajwan et al, 2019) in [8].

Once the uav deployment takes place and sensor nodes have been deployed routing in uav-wsn succeeds. Routing in uav assisted wsn is an important task that must be addressed. Sensor nodes that have been deployed will sense information from the surrounding and relay it to the control centers with the help of these uavs. The communication drains most the sensor node’s energy for which energy efficiency must be considered and routing protocols must be designed to keep the network alive for a longer duration.

Routing protocols for uav assisted wireless sensor networks can be categorized into three broad categories according to (Chan et al, 2020) [9]

**i**. Location based routing, **ii**. flat routing and **iii**. hierarchical routing techniques.

Location based routing focuses on the node’s current location with objectives to improve network scalability and to reduce routing overhead. In location aware routing our objective is to reduce the transmission of the control packets used in the routing process that are used by well know routing algorithms such ad hoc on demand distance vector AODV, dynamic source routing DSR etc. that flood the network with routing updates. A major drawback of location based routing such as location aided routing (LAR), distance routing effect algorithm for mobility (DREAM), AADTR, and earlier location based routing protocols is the use of flooding techniques to some extent in order to predict the next location of the destination node.

In flat routing algorithms a multi hop routing strategy is adopted by the nodes. Each node performs a similar type of task in order to construct a path towards the base station. when a node receives data packet it sends it to the next immediate neighbor in path towards the BS. The nodes have an identical role in the routing decision process. In flat routing protocols every node maintain an active path towards the base station and keep their routing tables updated due to which much of the energy of a node is depleted in performing these calculations. With large scale routing networks this aspect of flat routing become a serious issue. Scalability for network extension, load balancing are documented to be some of the issues in flat routing protocols.

In hierarchical routing nodes are grouped together in clusters. Clustering of nodes is performed by grouping nodes that have similar characteristics such as a common distance, certain attributed like node residual energy, urgency, received signal strength etc. The nodes are also divided into different roles according to these characteristics e.g. nodes with higher battery, processing power and are elected as the cluster head for a particular group. The remaining nodes are considered as cluster members. Non cluster members transmit data to their respective cluster heads. The cluster heads aggregate the data generated from different non cluster members and then transmit the aggregated data to the base station. At the beginning of each round the clusters head selection process is repeated.

Hierarchical routing protocols draw their foundation from the well known leach protocol which over the years different researchers have presented variations on its algorithm design which mostly focuses on resolving energy depletion issue to increase network lifetime. Large scale deployments in wireless sensor networks benefit more from the hierarchical routing as compared to flat or location based routing protocols. Recently surveyed by Khan et al. in [5] indicates that hierarchical routing techniques suffer from energy depletion due to complex procedures used in cluster formation, cluster head selection, identification of cluster members and non cluster member, route calculations, residual energy calculations, overcoming routing hole problems. Keeping in view of the above problems Khan et al. presented a dynamic priority based energy efficient hierarchical routing protocol most suitable for ad hoc wsn for measuring humidity, temperature from the agriculture farm lands. The calculations however are complex for an resource constrained wsn node to perform.

### Main research contributions

In this study a uav assisted wsn disaster zone is presented with. The initial objective will be to develop an efficient node deployment algorithm which is gateway based and ensures maximum connectivity of the user devices / sensor nodes (hereinafter referred to as sensor nodes) such that rejection of the sensor nodes is minimized. The node association will be on demand using the line of sight received power values for the association between the nodes and uavs which result in cluster like zone formation. The cluster formation will be the resultant of association between the nodes and uavs using the line of sight and non line of sight received signal power values rather the by using clustering specific algorithms such as k-means etc. Subsequently once the sensor nodes have been deployed and associated with the uavs the second phase will be to address the issue of energy efficient routing between sensor nodes and the uavs. Data transmission between the sensor nodes and the control centers drains most of the battery power of the nodes and hence a challenge for the routing protocol to preserve the energy of the network to increase the network lifetime. Contribution of this study are as follows:

**1**: Game theory based association of the deployed nodes and the servicing un manned aerial vehicles to establish hierarchical zones to be used by the EHGR routing protocol. In the game theory approach both the sides i.e. sensor nodes and the servicing uav’s optimize the functions to be associated only with the device that yields maximum functional values.
**2**: Game theory approach will associate only the nodes against which the optimization functions are generating maximum values. This means that the rejected node or the nodes that are out of the range of the uavs will not be able to communicate with the control centers. In the second part of the deployment phase we use gateway relay nodes to extend the network coverage for nodes that failed to get associated in the first phase. Relay nodes result in maximum coverage of the entire region.
**3**: The optimum deployment of nodes is passed to the next stage i.e. the energy efficient hierarchical gateway routing process. The EHGR divides the routing process in two phases zone formation phase and the data collection and routing phase which is usually called the steady phase.
**4**: EHGR run the energy centroid node algorithm on the uav’s to identify the zone heads hereinafter called the ECN nodes. The calculation of the ECN nodes are performed by the micro controllers embedded on the uavs to offload the sensor nodes from the extensive calculations which results in extended network lifetime.
**5**: In the second phase of the EHGR the ECN nodes accumulate the data and forwards the aggregated data to the servicing-uav instead of the control center. Again in this steady phase the much of the radio power is saved since the servicing uav is placed closer to the zone as compared to the control centers.
**6**: Energy dissipated during the routing process is calculated and the results show a substantial improvement in extending the network lifetime by 800 iterations such that the first dead node report occurs at the 979th iteration which surpasses the existing leach based approaches.

The rest of the paper is as follows. The ***related work*** section highlights latest scholarly work used for deployment and routing in uav-assisted wireless sensor networks. The ***energy efficient hierarchical gateway routing protocol*** section presents the network model for the study. In this section the detail working of the EHGR routing protocol is given. The ***simulation and results*** section gives detail discussion on various results and analyzes them against well know routing protocols. Finally in the last section the ***conclusion*** of the study is presented.

## Related work

6th generation and beyond networks address communication and energy issues of wireless sensor networks in emergency disaster regions to support time sensitive connections. Advancements in the 6th generation networks and beyond advocate ultra reliable and low latency use based data driven unlimited connectivity between sensor nodes, IoT smart devices, hand held user equipment maximized network throughput and abundance of energy [18] as pointed by (Lopez et al, 2021). These networks will allow sensor nodes to communicate with each other in an infrastructure less manner. Application requirements are increasing continuously such as use of interactive maps, video streaming etc. Supporting multiple sensor nodes/IoT devices to communicate and route data from one point to another in the wireless sensor networks the network layer continuously witnesses improvements.

UAV assisted IoT based networks have become a viable options for on demand routing between movable ground devices and non stationary nodes. Routing protocols have to be redesigned for uav-assisted wireless sensor networks that can accommodate continuous change due to high mobility and rapid topology changes.

### UAV assisted flat routing protocols

In flat routing nodes perform similar tasks and exchange similar information between nodes depending on the network architecture. (Liu et al, 2014) proposed an optimal distance based ant colony algorithm for routing in WSNs in [10]. Network lifetime is increased using the optimal transmission distance calculation using ant colony optimization algorithm. Initially all nodes are evenly deployed with the same amount of the battery power over a circular grid divided in omega sectors having a single sink node. Most energy efficient distance is calculated for multi hop environment that ensures maximum energy preservation. Data is collected by visiting different sites/nodes moving from once sector to another. However the protocol presented adopts a many to one transmission approach where in such scenarios a hot spot is created which can drain battery of the nodes residing near the sink nodes thus reducing network lifetime [19] (Lin et al, 2020).

(Brar et al., 2016) proposed pegasis dynamic source routing in [11]. The protocol reduces the communication distance using mutli dimensional transmission scheme which reduces energy consumption of the nodes. Nodes further preserve energy by generating a peering list against which no acknowledgment will be sent for received data packets against the listed peers. (Zeng et al., 2019) in [20] proposed a distance based energy efficient data collection flat routing technique that is tailored for uav-assisted wsn. The un manned aerial vehicles adopts a schedule of data collection from the sensor nodes. The uavs hover over the sensor nodes on a predetermined path to gather data for the active nodes and deliver it to a near by control server. A non-convex mixed integer approach is adopted by to determine the ideal path through an iterative algorithm. (Laouira et al., 2019) presented a flat communication multi layer approach for a uav assisted wsn in [21] to track human intrusions. Their work is divided into multi layer where the scalar sensor observe seismic activity and inform the second layer multi media sensors which use data fusion activities to inform the the nearby control center. The uavs are manually deployed once the human activity is observed however the energy of the uavs drain quicker as the number of intruder activities are increased as compared to the random deployment approaches.

### UAV assisted hierarchical routing protocols

In this approach the sensor network is divided into a hierarchical structure to establish clusters formation or tree like topology of the entire WSN. Tasks allocated to the sensor nodes can vary depending on the residual energy. Nodes having less energy can work as simply sensor nodes and the nodes having a specific power can perform multiple tasks such as data aggregation and also as a relay node. Table 1 highlights state of the art approaches for the UAV assisted wireless sensor networks. (Minhas et al., 2021) presented a hierarchical energy efficient routing inside a public safety network using only two uavs in [22]. A disastrous attack scenario is presented where the objective of the uavs is to carry user sensitive data either using uavs directly for communication within the cluster or by D2D gateway nodes in situations where the devices/sensor nodes lie outside the cluster head coverage to extend the coverage of the uav aided public safety network. The energy efficient routing algorithm proposed increased the energy efficiency by 15% as compared to traditional approaches.

**Table 1:** State of the art protocols summary with strength and weaknesses in each category.

Serial	Protocol	Category	Features	Observations
[10]	ACO	Flat evolutionary routing	Ant colony optimization distance based energy efficient routing. Multi hop relay.	Rapid creation of hotspots which increases routing holes visiting multiple sites on the optimized calculated route. Reduced network lifetime.
[11]	PDORP	Flat dynamic source routing. Hybrid of DSR & PEGASIS	Reduction of communication distance using multi dimensional transmission scheme which increases energy efficiency of the sensor network. Peer list generation to receive no acknowledgment packet to further improve node’s lifetime.	Protocol failure for alternate path calculation. Not suitable for dynamic real time situation. Calculation overhead to generate peer list.
[12]	Energy Optimization Algorithm	Flat routing for UAV assisted WSN border survalliance	Deployment of UAV to determine human border crossing. First layer of sensors sense human intrusion and second layer uses multimedia through UAVs to inform control center.	Rapid reduction for WSN lifetime as number of human intrusions increase.
[13]	TESEES	Hierarchical zone based energy efficient routing for heterogeneous network	Energy efficient routing IoT based wsn. Device makes decision to transmission using TMCCT algorithm to control un necessary drainage of batery power.	Relay node used for data transmission rather then network extension. Although network life is extended substantially as compared to leach based protocols but in real time ad hoc network this approach is not suitable as it ignore the coverage aspect.
[14]	EECRP	Hierarchical distance based energy centroid routing	Uses protection distance mechanism which saves energy for long distance data transmission.	Calculation of MAX distance depletes the nodes energy rapidly due to which the performance is slightly better as compared to LEACH.
[15]	IRP	Hierarchical progressive distance based routing	Threshold distance is used in which a node is allowed to send data directly to the sink node bypassing the CH.	The direct transmission generates routing holes rapidly.
[16]	GCEEC	Hierarchical distance gateway based routing	Selection of gateway nodes to reduce the burden of the CH. The nodes can use dual transmission by opting to send data directly to the ink node or use the CH. CH in turn uses the gateway node if the distance increases a threshold.	Computation performed by a sensor node are complex which reduce network life. Multiple CH in one zone which creates routing holes at a rapid pace. Since two cluster heads exist in every zone the interference at the MAC layer increases.
[8]	CAMP	Hierarchical zone based multipath routing	Use of IRP for non cluster members to save the load on CH from sending data directly to the sink node.	Energy calculations performed are complex due to which delay in the routing process is greater as compared to LEACH. Divides the network in equal size of cluster.
[17]	MEACBM	Multi-hop energy efficient routing protocol using multiple mobile nodes a hierarchical zone based energy efficient routing	Establish clusters and sub-clusters. Maintain network connectivity through multi hop relay for subclusters. MDC node is associated with each CH and calculates the optimum route towards the destination.	Establishment of sub-cluster drains th network energy rapidly and leads to reduced network lifetime.

(Shen et al., 2017) proposed ioT assisted wireless sensor network energy efficient routing protocols energy efficient centroid based routing in [14]. Shen et al. proposed a new concept called energy centroid which represents a location calculated by sink node in the wireless sensor network where concentration energy of sensor nodes is high. The sink node initially gathers the energy of the sensor nodes and the node closet to the centroid location is chosen as the cluster head. After each iteration of data transmission the cluster head calculates the node nearest to the energy centriod location and the new node is chosen as the cluster head. The protocol is self adaptive in the sense that after the initial selection of the cluster head through sink node the sensor nodes determine the next cluster heads locally to uniformly distribute energy depletion problem among the entire cluster thus increasing network lifetime. However it is observed that the concept of maximum distance consumes energy rapidly for the cluster heads due to caching and relaying data to the sink node.

Distributed clustering algorithm to establish cluster based on residual energy and distance of each node from the sink node is proposed by (Mahmood et al., 2018) in [12]. Cluster of different sizes are established by calculating RSSI values. Within the cluster node having highest residual energy is selected as the cluster head. The cluster size is kept minimum for the nodes laying near the sink node to preserve energy during realying and increasing the life time of the network. However it is observed that routing holes will be formed thus decreasing network life time. A new hierarchical intelligent routing process (IRP) protocol was proposed by (Sajwan et al., 2019) in [8]. Through the IRP a node can choose to send data directly to the sink node bypassing the cluster and this is done through multihop communication using the progressive node set. A node can cancel multi hop if the next hop selected towards the destination is a cluster member node in which the cluster head will be used to reach sink node. A distance threshold is defined that satisfies the transmitter free space energy and multipath energy consumption model which reduces the energy wastage. The proposed protocol improves network energy due this constraint by 970% as compared to the traditional leach algorithm. However it is observed that the energy calculation are too complex to be implemented in real time since node energy will be depleted quickly. (Naseer et al., 2020) in [16] proposed a new generation of hierarchical cluster routing protocol for wsn’s in the agricultural sector to monitor humidity, temperature and vital crop statistics. The algorithm uses gateway nodes for routing towards the control center and the routing is energy efficient and reduces the burden of the cluster head nodes. Initially a cluster is selected near the energy centroid location and then the gateway node within that cluster which is the edge node. The gateway nodes lye on the overlapping region within clusters en route towards the sink node. Similarly all the nodes in the overlapping region can be considered as gateway nodes however a node for which the node weight reaches a given value only that is selected. The nodes weight is based on the residual energy, distance from the neighbor cluster head and the distance from the sink node. For each iteration energy centroid location is calculated and the new cluster heads are selected along with new gateway nodes. This way the energy depletion is shared equally among the entire wsn. However it is observed that these calculation are far too complex for sensor nodes to carry on in a real time deployment scenario.

(Abdul-Qawy et al., 2021) in [2] proposed a multi level hierarchical routing protocol threshold oriented energy harvesting multi level stable election TEMSEP for large scale heterogeneous networks. Instead of continuous data transmission at regular intervals the TEMSEP protocol allows the node to respond only when the node senses a change in the data sensed. In TEMSEP a novel approach for sliding window concept is used which is reactive in nature and each node during its transmission time period will determine whether to transmit or remain asleep by examining a threshold. This method in which a node that not witnessed any change in the parameters of study will choose to keep its radio off thus increasing node lifetime. The energy harvesting nodes are deployed that provide intermediate forwarding service to the cluster heads to reduce the burden form the cluster head nodes. TEMSEP uses the first order energy dissipation model as proposed by Heinzelman in leach. The extensive experimentation indicates a 73% less energy dissipation and an increase by 69% in the network lifetime as compared to traditional hierarchical approaches.

(Abdul-Qawy et al, 2023) in [13] proposed an improvement over TEMSEP and proposed a reactive routing protocol to save the unnecessary data transmission which results in extending the node lifetime with in a each zone. The proposed routing protocol threshold enabled scalable & energy efficient TESEES. TESEES is a hierarchical zone routing protocol for large scale heterogeneous iot enabled WSNs. The protocol regulates the uplink data transfer in different zones against a threshold for controlling nodal energy. TESEES uses a sliding window in which every member within a zone can decide weather to transmit data or remain asleep by examining its threshold against the allocated time frame within the sliding window. The threshold algorithm TMCGT is deployed at each node individually and examines the past transmission event history to identify weather to transmit in real time against various parameters. TESEES divides the network according to different layers the second layer deploys relay nodes that harvest energy and forward the sensed data to the sink nodes. The initial network formation is divided into three phases static zones establishment, random node deployments and placement of relay nodes. Once the network is formed the weighted election heuristics algorithm is run for zonal heads called ZA’s nodes and the zonal aggregation group called ZAG. The ZAG election procedure is based on the MWEH algorithm which is based on multiple parameters as opposed to the traditional leacg based approaches. TESEES uses the TEMSEP thresholding algorithm to control the number of times a given node will be transmitting the sensed data. This approach limits the number of nodes within each zone that will keep their radio on or off according to the sliding window.

## Energy Efficient Hierarchical Gateways Routing (EHGR) protocol

A gateway based energy efficient routing protocol is proposed for ad hoc wireless sensor network in which a disaster region has been simulated. The use of gateways for routing has been proposed in [16] by (Naseer et el., 2020) and in [12] by (Mahmood et al, 2018) and by (Shen et al., 2017) in [14] but routing is primarily based on cluster formation in the network setup phase in which the sink nodes send location data to the sensor nodes which later on establish cluster heads. The cluster heads select member nodes and the selection of suitable gateways for each cluster is identified. Once the gateway nodes are determined by the routing algorithms they are fixed for all the rounds within the routing and data transmission process. However in disastrous regions and ad-hoc wireless sensor networks prior knowledge about placement of gateway nodes on the network grid can not be predicted in advance and hence the routing protocol must re evaluate the selection of gateway relay nodes in each round and reconfigure the entire zone by calculating new set of gateway nodes as the routing protocol progresses further. The proposed routing protocol EHGR is completely random in terms of node deployment and the selection of gateway nodes. EHGR adopts game theory approach in the first phase to dynamically associate nodes with the servicing-uavs using the matching theory algorithm which results in zone formations used by the next phase of the routing protocol. The game matching algorithm is different from traditional cluster based algorithms such as k-means etc. where a single head node makes all the decision against a given cluster. In the game theory both the nodes and the servicing-uav run the matching algorithm to maximize their objective function values. Each side running the matching algorithm is associated only with the other side having maximum values of the objective function which results in a dynamic zone formation of the entire network. In the first phase of EHGR after the nodes are associated with the servicing uavs by running the game matching algorithm the selection of gateway relay nodes will be performed to extend the coverage of the network for all those nodes that have failed to get associations from any of the servicing-uavs. The gateway nodes used for d2d data forwarding minimize the rejections of the nodes that have data to transmit to the control center.

EHGR helps to overcome an prominent networking problem highlighted in the literature of the hierarchical routing algorithms which is the energy hole problem. Nodes acting as relay nodes and cluster head nodes drain their battery power quickly as compared to the rest of the nodes and due to this these nodes die out fast. The dead nodes create hole and routing becomes difficult since these hole in routing data through network cause isolated groups of nodes that can not communicate with each other as pointed out by (Arfat et al., 2020) in [23]. Arfat et al tackled this problem and their proposed protocol that selects a different gateway node after each iteration by adding a weight function that increases the weight each time a gateway node is selected thereby decreasing the likelihood of the same node elected for relay purpose. The EHGR protocol places the burden of energy centroid nodes selection, zone formation and data forwarding to the servicing-uavs which result in gradual energy dissipation thereby extending network life. EHGR offloads the zone heads against computationally intensive operations due to which a node’s active lifetime is increased. The depletion of the energy within network is gradual in comparison to the existing routing protocols deployed in ad hoc wsns. In the data aggregation and transmission phase again EHGR performance is substantial as the energy centroid node of each zone will offload its aggregated data to the servicing uav hovering right above it. In hierarchical routing protocols the cluster heads use a direct communication technique to transmit the aggregated data to the sink nodes due to which early routing are generated.

### Network setup phase

In the disaster region UAVs have been considered to be ideal due to their ability of providing cellular and data services. The UAVs can increase the productivity of WSN in terms of data communication and aggregation among the nodes. Use of UAVs for routing in wsn is an emerging domain with scarcity in terms of the available literature for uav assisted routing protocols. UAVs are used in [15] by (Ebrahimi et al, 2018) to gather data from cluster heads in sensor networks and route it to the nearby sink node for data processing thus increasing network lifetime. The entire network is arranged into hierarchical clusters and the path that the uav will travel is analyzed in prior using the compressive data gathering approach. (Ho et al., 2015) in [24] proposed a uav based energy efficient communication topology to reach destination with reduced bit error rate as compared to leach protocol. Swarm intelligence is used to figure out the path that reduces the uav travel length with reduced bit error rate. (Baek et al., 2019) proposed an energy efficient routing in uav assisted wsn for data collection in [25]. Energy maximization against residual energy is achieved by using the graph based voronoi diagram to identify the shortest distance during routing process. Once the shortest path is calculated the uav hovers to the target location to gather data coming from multiple sensor. UAV assisted wsn offload the burden of cluster heads during data aggregation and increase network life time by delivering the data to the nearby control centers.

In this study unmanned aerial vehicles are deployed over the disaster region. Game theory is used to associate nodes using the received signal strength indicator (RSSI) and for selection of gateway node. Model for energy consumption is derived in this phase.

Initially K sensor nodes are deployed on a simulated gird. We assume a control center with a configured location that will act as the last establish point beyond which there is no established network infrastructure.

The sensor nodes are randomly deployed on the network area.

Let K be set of sensor nodes also referred to as user equipment. $\{k\in Sn_{p}{\}}_{k=1}^{n}$

Each *Sn*_*p*_ is aware of its GPS location relative to the network. Initially a node from set (*Sn*_*p*_) receives line of sight and non line of sight transmission signals generated from the un manned aerial vehicle. Line of sight (LoS) and non line of sight (NLoS) are the received signal a nodes calculates from a direct link from the UAV and non line of sight is the signal value a node receives indirectly by reflections, refraction, free space signal shadowing etc. These values were initially pointed out by (Al-Hourani et al., 2014) in [26] for low altitude platforms and can be approximated by the sigmoid function as

$$\begin{array}{c} P\left(LoS,\Theta \right)=\frac{1}{1+\alpha .e^{\left(-\beta (\Theta -\alpha )\right)}} \end{array}$$ (1)

and

$$\begin{array}{c} P(NLoS,\Theta )=1-P(LoS,\Theta ) \end{array}$$ (2)

here *α*, *β* represent the built land and average buildings in respect to total deployment area respectively. We use both the values in our energy model. Θ is the elevation angle according to Al-Hourani et al. and can be expressed as Θ = *arctan*(*h*/*r*_*kn*_) where h is given UAV altitude and *r*_*kn*_ is the ground distance from each user equipment /sensor node *Sn*_*p*_ with the UAV. Table 2 summarizes the parameters and the notations used for establishing the network model. The sensor node will initially be associated with one UAV over this path in the first phase to become a member of a zone. In the association phase each sensor node receives a hello message from the UAV either through line of sight or non line of sight. Each sensor node will calculate the achievable data rate from each UAV before placing a request for association which is given by

$$\begin{array}{c} \begin{array}{c} A_{dr}=P\left(LoS,\Theta \right)*(20log\left(d\right))+20log\left(f\right)+20log\left(4\pi /c\right)+\eta_{LoS})+\\ P\left(NLoS,\Theta \right)*(20log\left(d\right))+20log\left(f\right)+20log\left(4\pi /c\right)+\eta_{NLoS}) \end{array} \end{array}$$ (3)

**Table 2:** Parameter symbols with notation interpretation.

Parameters	Notation
*P*(*LoS*, Θ)	Probability of received signal at a sensor node from a given servicing uav at line of sight with an angle theta
*P*(*NLoS*, Θ)	Probability of received signal at a sensor node from a given servicing uav at non line of sight with an angle theta
*A*_*dr*_	The total data rate of any given node with a given uav
*Cls*_*Throughput*_	Throughput of one zone of a given servicing uav
$\frac{\sum_{i=0}^{n}\frac{E_{residual}i}{E_{initial}i}.X}{N}$	Residual energy of the i node divided by the initial energy of the i node. Multiplied by the x-coordinates of the ith node.
$\frac{\sum_{i=0}^{n}\frac{E_{residual}i}{E_{initial}i}.Y}{N}$	Residual energy of the i node divided by the initial energy of the i node. Multiplied by the y-coordinates of the ith node.
$\left(\bar{x_{ec}},\bar{y_{ec}}\right)$	(x,y) coordinates of the energy centroid nodes
*E*_*Tx*,*d*_(*l*, *d*)	Energy required to transmit l bits of data a distance d

**Fig 1:**
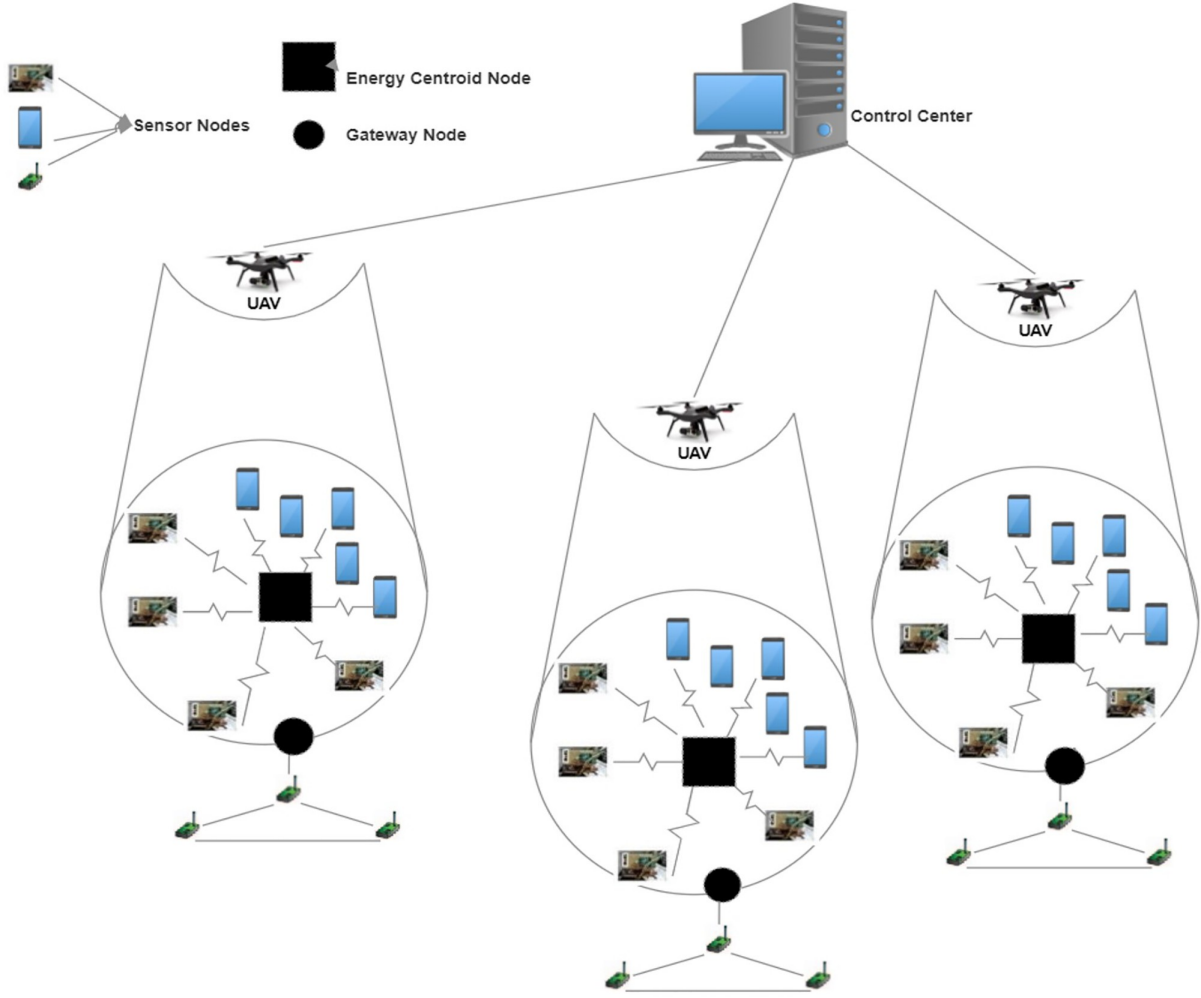
Network model with energy centroid and multiple gateway nodes.

The same UAV which will select a suitable energy centroid node in the next phase from these associated nodes in the network. Here d is the distance between sensor node and the UAV having projection on ground as shown in Fig 1 which is calculated by *d* = *sqrt*(*h*^2^ + *r*^2^) *f* is the given frequency.

The total achievable rate is the throughput of the cluster from a single UAV which is given as

$$\begin{array}{c} \begin{array}{c} Cls_{Throughput}=\sum_{k\epsilon K}^{}A_{dr},ifk_{th}\;\;node\;connected\;with\;i_{th}\;UAV\\ 0,otherwise \end{array} \end{array}$$ (4)

#### Node association & ECN-energy centroid node selection phase

From the network model presented in Fig 1 can be seen that sensor nodes that are in the range of a particular uav will be grouped together to establish a zone formation. The zone formation here is established with the help of a game theory matching algorithm approach. In the game theory approach the routing is based on cost and payment model where both sides try to maximize the gain. In the association phase of the EHGR both the parties i.e the sensor nodes and the unmanned aerial vehicles have an objective to maximize their objective function values which results in maximized coverage, minimized rejections and better throughput which means that the sensor nodes will try to associate with the servicing-uav from which maximum *A*_*dr*_ is calculated and similarly the servicing-uavs will associate only those nodes from which highest line of sight and non line of sight signal values are received. The equations presented in Eqs 5, 6 and 7 are performed in parallel by the nodes and the servicing uavs in the first phase of the EEHGR.

The objective function is to

$$\begin{array}{c} max_{A_{dr}}Cls \end{array}$$ (5)

$$\begin{array}{c} \sum_{n\epsilon N}^{}\leq 1\;\exists \;\;k\;s.t\sum_{k\epsilon K}^{}A_{dr}\;>0 \end{array}$$ (6)

$$\begin{array}{c} \sum_{k\epsilon K}^{}A_{dr}\leq B_{bandwidth}\exists \;\;n\epsilon N\;\;s.t\;\;B\leq C_{o} \end{array}$$ (7)

As per Eq 5 the objective is to maximize the throughput with in each cluster. In Eq 6 suggests that a sensor node whose *A*_*dr*_ value is greater than zero can be associated with a single UAV or not associated at all. Similarly in Eq 7 it is clear that achievable rate can not exceed the channel capacity which sets an upper bound on the used bandwidth. The above equations can now be taken as an optimization problem which is non linear in nature. The association algorithm presented here addresses the game theory approach from both parties.

**Node Association Algorithm**:

         **Algorithm for Node Association**

**1. Input Data: K, N, B**

**1. Output**: UAVs associate k nodes to maximize *A*_*dr*_ against total available bandwidth B

**2. Target: Max. Node Association**

**3. Vector Matrix Initializations**: Sk, Matrix of all k belong to K node, Sn, Matrix of all n belong to N UAVs

**Initial Deployment phase**

**4**. Each node k belong to K generates a list of each UAVs with in distance ‘r’

**5**. Bn total bandwidth of the UAV or the resources

**Application of the UAV and the sensor Node using Game Theory**

**6**. Each UAV in n belonging to Sn sends a HELLO message

**7**. Sensor nodes prioritize UAVs according to the Adr values of each UAV

**8. while (Sk matrix is !=0)——Max B is for each UAV is achieved**

**9**. Each sensor node k belonging to Sk sends the association request to UAV in Sn from which it get highest Adr

**10**. Each UAV n in Sn prioritizes in reverse the k nodes in Sk according to the Adr

**11**. Each UAV n associates the node that receive highest *A*_*dr*_ value from it

**12**. Once the sensor node is associated with a UAV it is removed from the list of nodes in the Sk matrix

**13. End while**

In order to save the energy of the sensor nodes initially establishing the clusters and further selection of the energy centroid the servicing-uavs initiate a HELLO message formation as shown in table labelled HELLO message format. each entry of the HELLO message is one byte in length. The sensor node use this HELLO message to rank all the UAVs accroding the their respective *A*_*dr*_ data rate.

**HELLO message format**

MessageType|SenderID|X-Cordinate|Y-Cordinate

from line 8 on wards in the node association algorithm the end of the while loop two different phases take place one in which sensor nodes rank the UAVs according to the highest data rate and second in which UAVs also examine the *A*_*dr*_ values of the requesting sensor nodes to associate the best candidates and reject the rest. Once each node has been associated a Feed Back reply is generate from each UAV shown in table labeled Feedback reply message from UAV and sent to all associated sensor nodes in one cluster. Servicing UAV-ID will be used for D2D forwarding for network extension. Once the energy centroid node is calculated it will send a join request to all the member nodes to establish link in the cluster.

**Feedback reply message from UAV**

MessageType|Energy-CentroidNode ID|ServicingUAV-ID|AverageEnergy

As in Fig 1 it can be seen that the concentration of the sensor nodes in each zone formation is biased having dense deployment in one zone and sparse in the other. This concept is used by (Jian Shen et al., 2017) in [14] in which energy centroid node is calculated for each cluster and the node nearest to the energy centroid is taken as the cluster head or the energy centroid node (ECN). This energy centroid node becomes the zone head which aggregates data from the zone member nodes and forward it to the servicing-uav which will route it towards the control center.

$$\begin{array}{c} \bar{x_{ec}}=\frac{\sum_{i=0}^{n}\frac{E_{residual}i}{E_{initial}i}.X}{N} \end{array}$$ (8)

$$\begin{array}{c} \bar{y_{ec}}=\frac{\sum_{i=0}^{n}\frac{E_{residual}i}{E_{initial}i}.Y}{N} \end{array}$$ (9)

#### Gateway node selection for D2D multi hop relay

In uav-assisted wsn selection of gateway nodes can improve the coverage and network throughput. Gateway nodes increase the network connectivity reducing the number of rejected nodes. UAV coverage might be partial where some of the sensor nodes are not be in the coverage zone due to physical terrain due to which those nodes can only route data towards the control center through gateway nodes as pointed out by (K. Ali et al, 2017) in [27].

**Data transmission and energy centroid rotation algorithm**

**Algorithm for data-transmission & ECN-rotation for each round**

***Phase-I***

**for j = 1 : k s.t. k** ∈ **to K node set**

each k node having l bits to transmit

**if (j ==cluster member)**

 *E*_*r*_*l* = *E*_*r*_*l* − *E*_*T*_*l* − *ϵ*_*fs*_*l*

 /*residual energy *E*_*r*_ updated at each transmission */

**else-if (j==ECN){**

 ***do***

 **{**

  *E*_*r*_*l* = *E*_*r*_*l* − *E*_*R*_*l*

 ***while (data signal is sensed on receiver)***

 *E*_*r*_*l* = *E*_*r*_*l* − *E*_*T*_*l* − *ϵ*_*fs*_*l*

  **}**

**for i = 1** : *k*_*m*_

Each cluster member node (*k*_*m*_) send location & residual energy *E*_*r*_(*k*_*m*_)

to the servicing UAV

**end-for**

***Phase-II***

Servicing-UAV calculates average energy of the cluster

$E_{o}=\sum_{n=1}^{k_{m}}\frac{E_{r}n}{k_{m}}$

servicing UAV updates the energy centroid

$\bar{X_{ec}}=\sum_{n=1}^{k_{m}}\left(\frac{E_{r}n}{E_{o}}.X_{n}\right)/k_{m}$

$\bar{Y_{ec}}=\sum_{n=1}^{k_{m}}\left(\frac{E_{r}n}{E_{o}}.Y_{n}\right)/k_{m}$

$d=\sqrt{{\left(\bar{X_{ec}}-X_{n}\right)}^{2}+{\left(\bar{Y_{ec}}-Y_{n}\right)}^{2}}$

**for k = 1** : *k*_*m*_

**if** (*E*_*r*_*k* > *E*_*o*_ && *d*_*km*_ **is least** ∀ *k*_*m*_ **nodes**)

**{**

 send Feedback packet to km cluster members.

**} end-for**

ECN node sends join request to all *k*_*m*_ members.

**end-for main loop**

**Cluster formation by member nodes and ECN node**

**Algorithm for cluster formation after the feedback packet**

Servicing-UAV sends Feedback message to all *k*_*m*_ cluster member

**for i = 1**: *k*_*m*_

each i node examine the node ID

**if (Node(i).ID == ECN.ID)**{

 Mark as the ECN & Open receiver antennas

 Send Join request packet to the cluster members

**}**

**else-if(Node(i).ID != ECN.ID)**{

 Wait to receive join request packet

 update the ECN-ID for transmission

 send join reply

 data transmission phase

**}**

**end-for**

**Fig 2:**
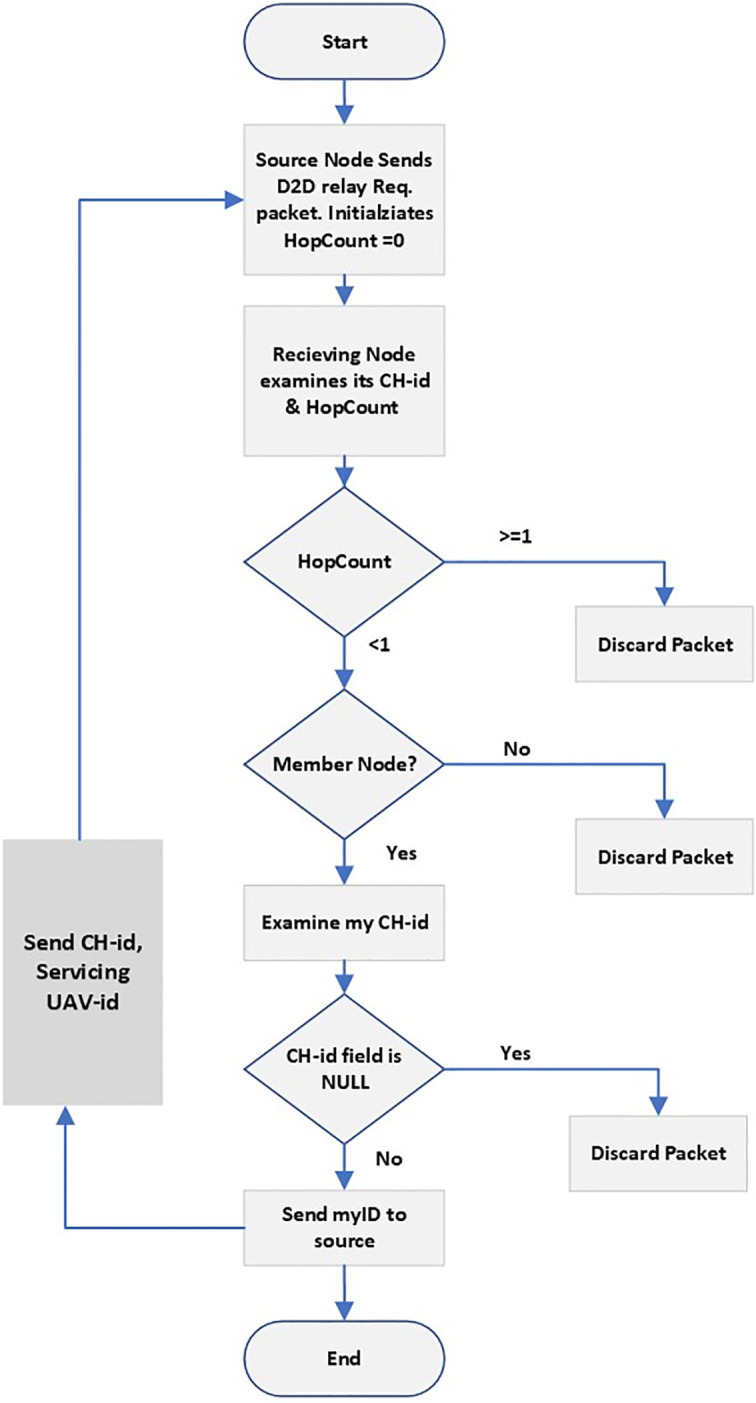
D2D routing for relay node.

Nodes that have not received the Hello and the feedback message from the servicing-uav but overheard the acknowledgment to the join request sent from the centroid node will be connected to the same zone using the D2D gateway relay forwarding these are the green nodes if we examine Fig 1. D2D gateway relay node will help to minimize the rejected nodes thus increasing network connectivity. From Fig 1 each green colored sensor node will send a D2D relay request packet to the neighboring node which has already been associated and whose acknowledgment was just overheard. In this study one hop relay request will be accepted. D2D schematic flow diagram is presented in Fig 2.

If the receiver node that receives a relay request packet finds that the hop is greater then 1 it discards since 1 hop relay is considered in this study. Seminal study for energy aware wsn conducted by (Heinzelman et al, 2000) in [28] mathematically proved that direct transmission to the receiver gateway dissipates less energy as compared to multi hop. Similarly if the cluster head id of the energy centroid node is missing then it discards the packet since the receiver is also unaware of the gateway node and is also in search for it thus hop limit will be greater than 1 again. The nodes lying outside the circular grid will use use D2D forwarding techniques such as shortest path routing, flooding neighboring approach etc. to reach the gateway node which will forward the data collected to the energy centroid node in the respective cluster. The energy centroid node will forward the data to the control center through servicing un manned aerial vehicle.

This study uses the first order energy model proposed by Heinzelman et al in [28]. According to Heinzelman the energy required to transmit l bits of data at d apart

$$\begin{array}{c} E_{Tx,d}\left(l,d\right)=E_{Rx,d}\left(l,d\right)=E\left(l,d\right)=\{\begin{array}{c} l(e_{r}+e_{t}+\epsilon_{fsd^{2}}) \end{array} \end{array}$$ (10)

In this model *l* is the packet length in bits, *e*_*r*_ and *e*_*t*_ are energy spend by a node during transmission and receiving over distance *d* with the UAV and its self.

#### Data transmission & energy centroid rotation phase

Examining the data transmission and energy centroid rotation algorithm once the optimal deployment of the sensor nodes is accomplished data transmission begins. Sensor nodes send data to the energy centroid nodes (ECN) which forward it to the servicing un manned aerial vehicle. The servicing uavs forward the aggregated data to the control center. After each successful transmission the ECN nodes examine the residual energy and if it falls below a threshold an ECN transfer request is initiated to the servicing UAV. The UAV recalculates the average energy of the cluster to select a new ECN. The ECN sends a new join request to the member nodes.

The data transmission round in each cluster is divided into two phases. Initially every node within a zone having data to transmit will transmit the data to the ECN node following the scheduling time window. During transmission the energy dissipated is equal to the subtraction of the energy required during transmission of *l* bits *E*_*T*_*l*, the transmit amplifier energy $\epsilon_{fsd^{2}}$ from the node’s residual energy *E*_*r*_. When receiving the *l* bits packets the energy required only subtracts the receive energy *E*_*r*_*l* for *l* bit and the amplifier circuit energy $\epsilon_{fsd^{2}}$ is excluded. In data transmission and ecn rotation algorithm presented *phase*–*I* node residual energy during transmission and the residual energy of the ECN node for accumulating data is calculated. Just before finishing this phase all nodes within a zone send residual energy and location to servicing uav for calculating energy centroid node for the next round. In the earlier hierarchical routing protocol the energy lost due to computations performed by the cluster head node that places additional burden on the cluster head node which results in reduced network lifetime and early dead node detection. However in this study just before the termination of each round the residual energy is sent to the servicing uav which offloads the existing zone head nodes for performing these calculations for the next round. This approach extends the network lifetime since the computationally intensive operation against each round is shifted to the servicing-uav which further extends the network lifetime and delays the detection of the early dead nodes.

In the second phase of the data transmission algorithm the servicing UAV calculates the energy centroid for the entire zone by considering the residual energy *E*_*r*_ reported by each node from the *k*_*m*_ nodes. Once the new ECN is calculated the servicing UAV sends feedback packet to the nodes (the algorithm labeled cluster formation by member nodes and ECN node). The new ECN node will send join request to all the zone members to which the member node reply by acknowledging the join packet. All nodes with in the cluster examine the node id with its own id. If the node id matches with the id sent in the feedback message the node marks its self as the ECN node and sends the join request. Otherwise the node with different id waits for the join request from the energy centroid node to become a member node.

## Simmulation and experimental results

The experimental setup is conducted using matlab with parameter values shown in Table 3. The network grid is established after which 200 sensor node are randomly deployed. The nodes are heterogeneous in nature. Work presented in [27] by (K. Ali et al, 2017) [16] by (Naseer et al., 2020) [8] by (Sajwan et al, 2019) [29] by (Yao et al., 2022) focus on ad hoc deployments of wireless sensor networks in which the number of nodes are assumed to be 100 but the work presented in this study considers a heterogeneous group of nodes twice the size of the current studies and the reason for this is that these techniques mentioned above focus on either the optimized coverage or the routing of data to the control center or data offloading by the un manned aerial vehicles but not all in parallel. The network grid measure 400*m*^2^. Location of the control center as from Fig 1 is outside the network that can be accessed through the aerial drones.

**Table 3:** Parameter values used in simulation.

Simulation Parameters	Values
Network area	400 *m*^2^
Control Center	Outside network grid
Sensor Node	200
Initial Energy	0.5 J
Data Aggregation energy	5 nJ/bit/signal
Transmit Energy	50 nJ/bit
Receive Energy	50 nJ/bit
*ϵ*_*fs*_	10 pJ/bit/*m*^2^
*ϵ*_*amp*_	0.0013 pJ/bit/*m*^4^
Packet size	200 bit
Bandwidth availability per UAV	10 Mbps
UAVs availability	Varies with number of Clusters
*α*, *β*	9.61, 0.16

### Network extension and optimized coverage during deployment phase-I in EHGR

**Fig 3:**
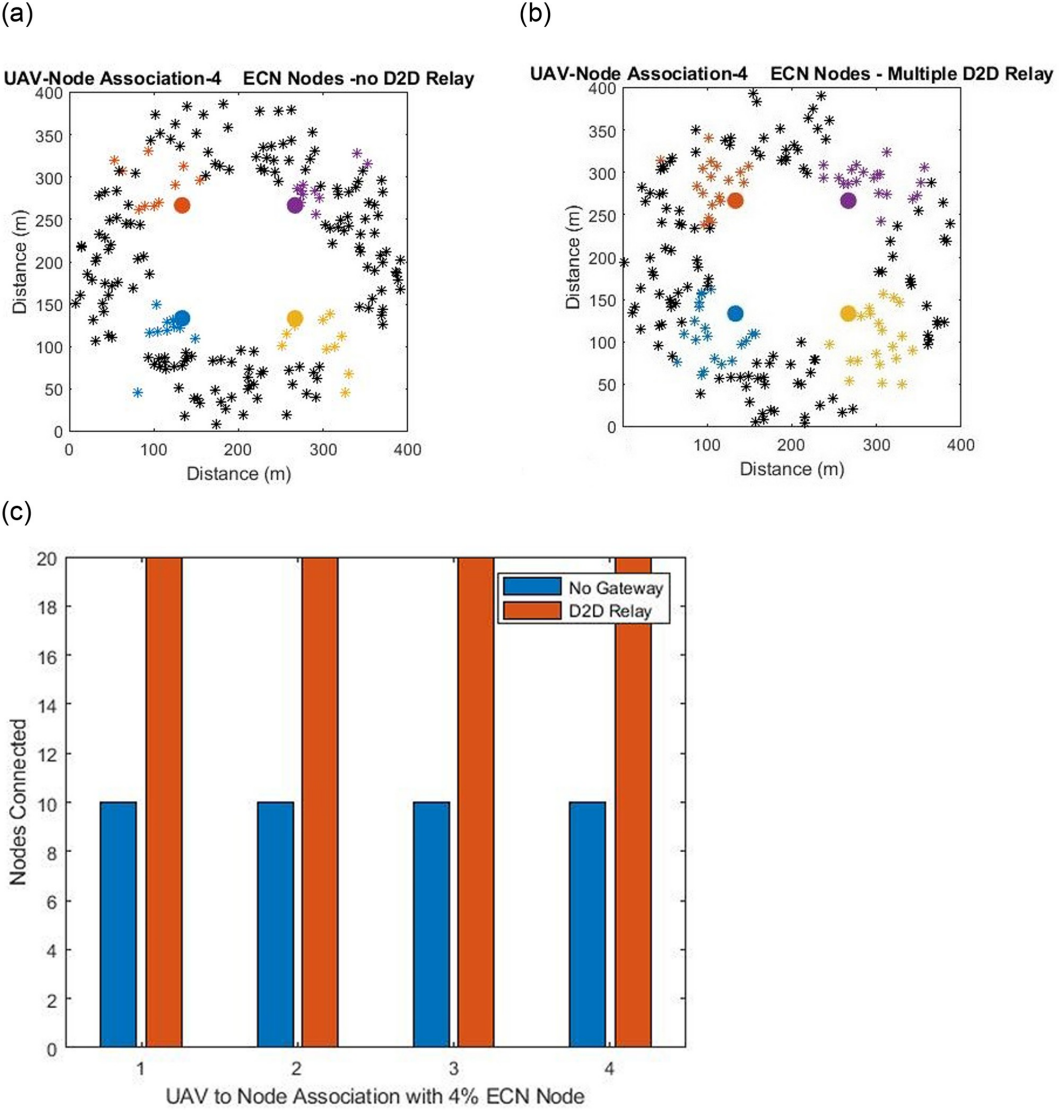
(a). Deployment with 2% ECN Nodes with no D2D Gateways Relay Node (b). Deployment with 2% ECN Nodes with relay Gateway nodes (c). Comparison between 2% ECN node with and without relay nodes.

**Fig 4:**
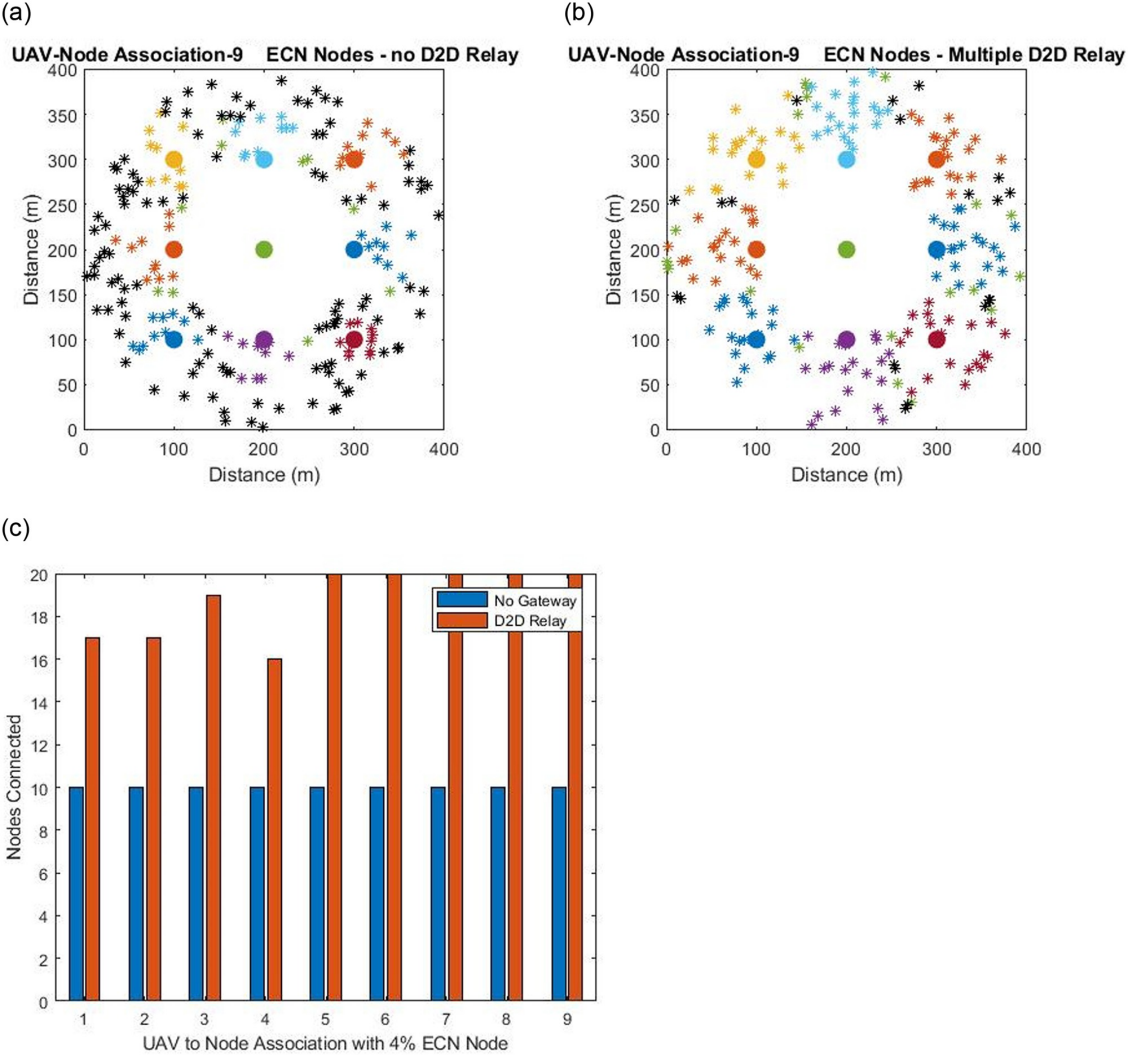
(a). Deployment with 4.5% ECN Nodes with no D2D Gateways Relay Node (b). Deployment with 4.5% ECN Nodes with relay Gateway nodes (c). Comparison between 4.5% ECN node with and without relay nodes.

During the deployment phase of the network nodes are randomly scattered on the entire network grid. Some the nodes get placed on the edges due to which it is not possible to obtain a complete coverage scenario. The nodes once deployed run the game theory matching algorithm along with the servicing aerial drones. The outcome of the matching algorithm is that aerial drones associate maximum number of nodes against the available bandwidth. The connectivity at this stage of the deployment process will have a large number of number of nodes that have failed to get any association from any of the servicing drones. Some of the nodes can be at edge of the network that fail to get association from any servicing-uav. Some of the nodes might be within the coverage zone of a particular drone but due to the limited bandwidth capacity available on the micro controller which is 10 mega bytes failed to get associated. To increase the connectivity beyond this point gateways nodes have been proposed. The gateways nodes will associate the nodes that have not yet been till this point and thus the gateways will route data from these nodes to the respective energy centroid node from which the gateway node belongs. In this study only one hop gateway node extension is assumed and in the future works multi hop gateway connectivity will be considered which will result in even more nodal coverage. The work presented in earlier studies of ad hoc wireless sensor networks assume connectivity scenarios where the number of cluster heads with in the network range from 2% (two cluster heads) and 5% (5 cluster heads) but since in this study the number of nodes have doubled therefore we have kept the ratio approximately same by using 2% and 4.5% ECN nodes as can be seen in Figs 3 and 4.

Examining the first top left sub figure presented in Fig 3 having 2% ECN nodes. In the diagram a node is represented by a cross. The nodes that are represented as black crosses have failed to get any association and the color cross nodes match with their energy centroid nodes represented by a bold color circle. The deployment scenario after the game theory matching algorithm is run and the number of nodes that failed to get any association is large. To increase the connectivity beyond this point gateways nodes are deployed and the right sub figure presented in Fig 3 shows the mapping of the nodes. The number of connected nodes have doubled as compared to left sub figure and this is due to the gateway nodes. However it can be observed the still there are some nodes that have failed to get any association further. As pointed out earlier that this can be resolved by adopting a multi hop connectivity schemes such that the down link (nodes away from the energy centroid nodes and the control center) group of nodes are able to connect through gateway nodes thus making a chain like resemblances to gain even higher coverage impact. The exact number of connectivity in both the scenarios is present in the lower sub figure of Fig 3. The nodes that are not associated in with the energy centroid nodes will thus use D2D forwarding techniques to transmit the information to the control centers.

Fig 4 represents the scenarios of deployments with marking 4.5% of energy centroid nodes. However in the second case of the deployment experiment number of nodes that are connected in the network after the game theory matching algorithm is double to that of Fig 3 and the reason is due to increased number of energy centroid nodes which result in higher mappings. In Fig 4 when the gateway nodes are deployed the number of connected nodes doubles. However again it can be observed that some nodes have failed to get any association. The nodes that fail to get any association will become part of the network again in the next iteration process when the routing phase starts.

From the deployment experiments in Figs 3 and 4 it can be seen that the node deployment of the ad hoc wireless sensor network can be extended to a maximum limit of the available bandwidth by using efficient deployment and gateways nodes for network coverage. In both the cases presented above the result of deploying D2D gateway relay nodes outperform the cases with no D2D gateway relay nodes. However maximum network extension is not reached due to one hop restriction and second that even some of the nodes are outside the coverage zone of the cluster nodes and miss the acknowledge packet sent by the cluster member nodes to the ECN nodes for networking joining as shown in the D2D relay gateway flowchart in Fig 2. That is why in the case where 9% ECNs nodes based clusters are established some of the nodes are outside all the coverage from the member nodes.

### Routing phase and data transmission to the control center phase-II in EHGR

Once the deployment scenario is optimized routing process begins. The first iteration of the routing processes uses values passed down from the deployment layer that indicates the initial energy centroid nodes and the servicing UAV’s. Each node follows a transmission schedule generated form the energy centroid node. Every round within each iteration calculates the energy centroid node. ECN node calculations are performed at the micro controller enabled servicing uav’s which offload the computations carried out at round. The energy dissipation model calculates the energy dissipated as presented in the routing algorithm for data-transmission & ECN-rotation during the transmission phase and reception phase, data aggregation phase of the nodes and energy centroid nodes. In the work done by Naseer et al in [16] relay nodes are used to transmit data to the control center to preserve energy dissipation at the cluster head nodes. But this issues generates routing holes rapidly such that the burden placed on the relay nodes doubles since the relay node will receive the transmission from the cluster head node and use its own radio to transmit the data to the control center. This decreases the network lifetime by doubling the ratio of the dead nodes. However in EHGR the data is offloaded by the servicing UAV which limits the use of relay gateway node and again improves the network lifetime.

In energy efficient routing protocols such as in [15, 16, 24, 30], LEACH and its variants much of the node energy is dissipated towards the end in each iteration when the entire cluster recalculates the new cluster head, member nodes association with the cluster head, cluster head reconfirmation to the joining nodes, which decreases the network lifetime and creates routing hole quickly.

**Fig 5:**
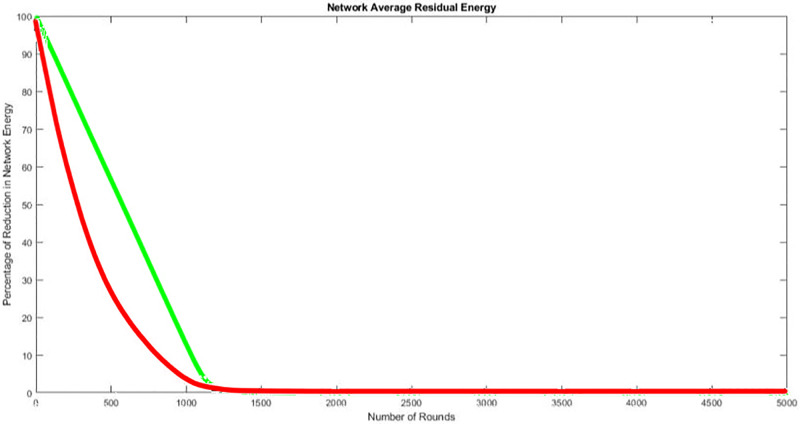
Calculation in the reduction of energy after each round.

Examining Fig 5 which shows the average residual energy of the entire routing process for leach in red and EHGR in green during all the phases it can be observed that the methodology adopted in the EHGR protocol to preserve the network energy is stable and effectively the entire network remain above 50% total average energy even after 500 iterations as compared to the leach protocol. This is due to the fact that computationally intensive calculations in deployment phase, ECN node selection phase, transmission of data to the control center phase are offloaded to the servicing UAV’s thus increasing the network lifetime. Therefore it can be concluded that the energy dissipated is reduced by over 100% as compared to leach protocol.

**Fig 6:**
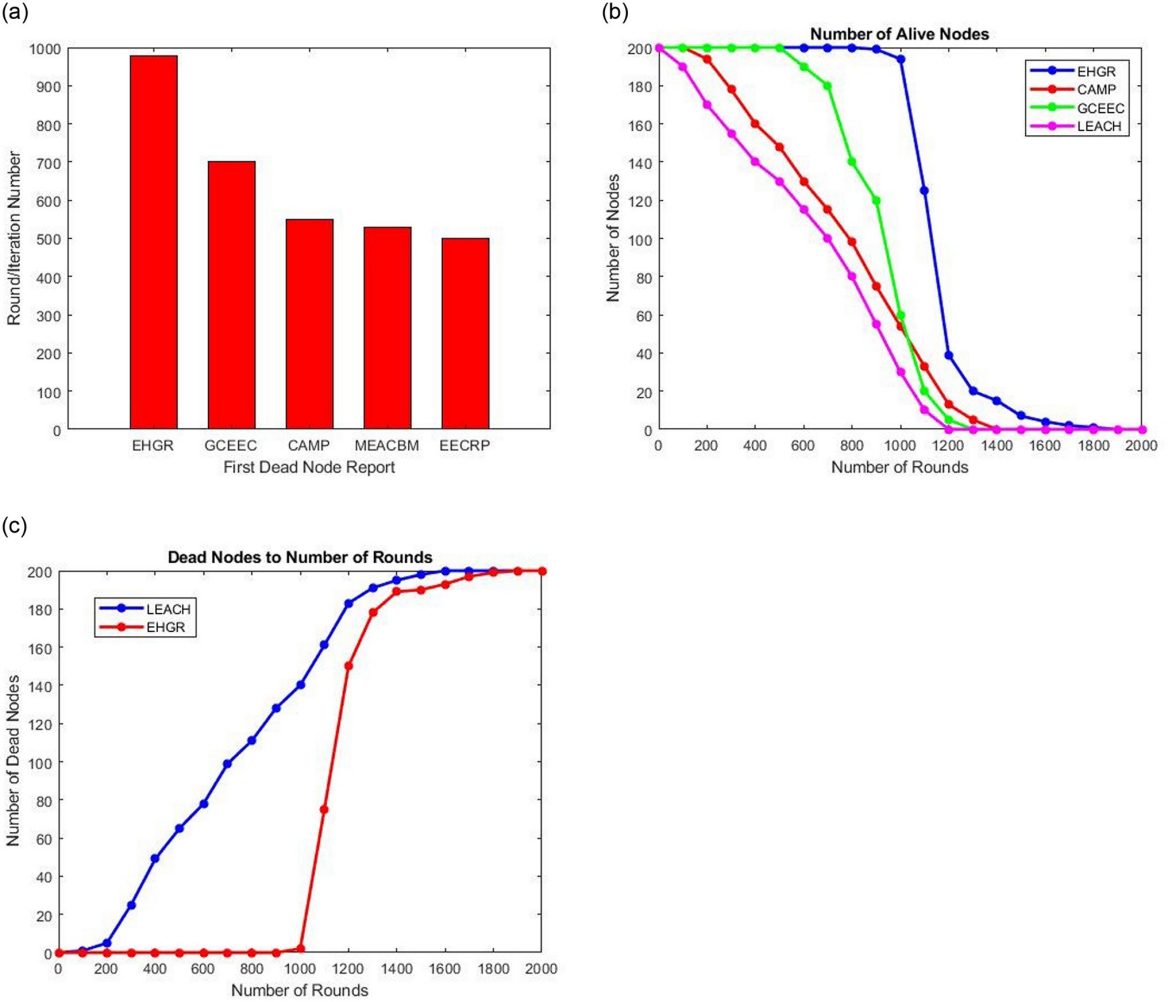
(a). First Dead Node Report (b). Decrease in Number of Alive Node during each Iteration (c). Increase in Number of Dead Nodes during each Iteration.

Examining Fig 6(a) the bar chart for the first dead node report is presented which can bee seen to substantially be reported at longer interval of rounds which shows the network lifetime has been extended. Fig 6(b) shows the decrease in the number of alive nodes during each rounds. In Fig 6(b) it can be seen that EHGR outperforms the existing hierarchical routing protocols for wireless sensor networks due to it data and computation offloading capabilities to the servicing uavs. In Fig 6(c) the increase in the number of dead nodes can be observed against each round. From all three sub figures it can be observed that the active network lifetime is nearly 1800 rounds.

#### Network throughput

**Fig 7:**
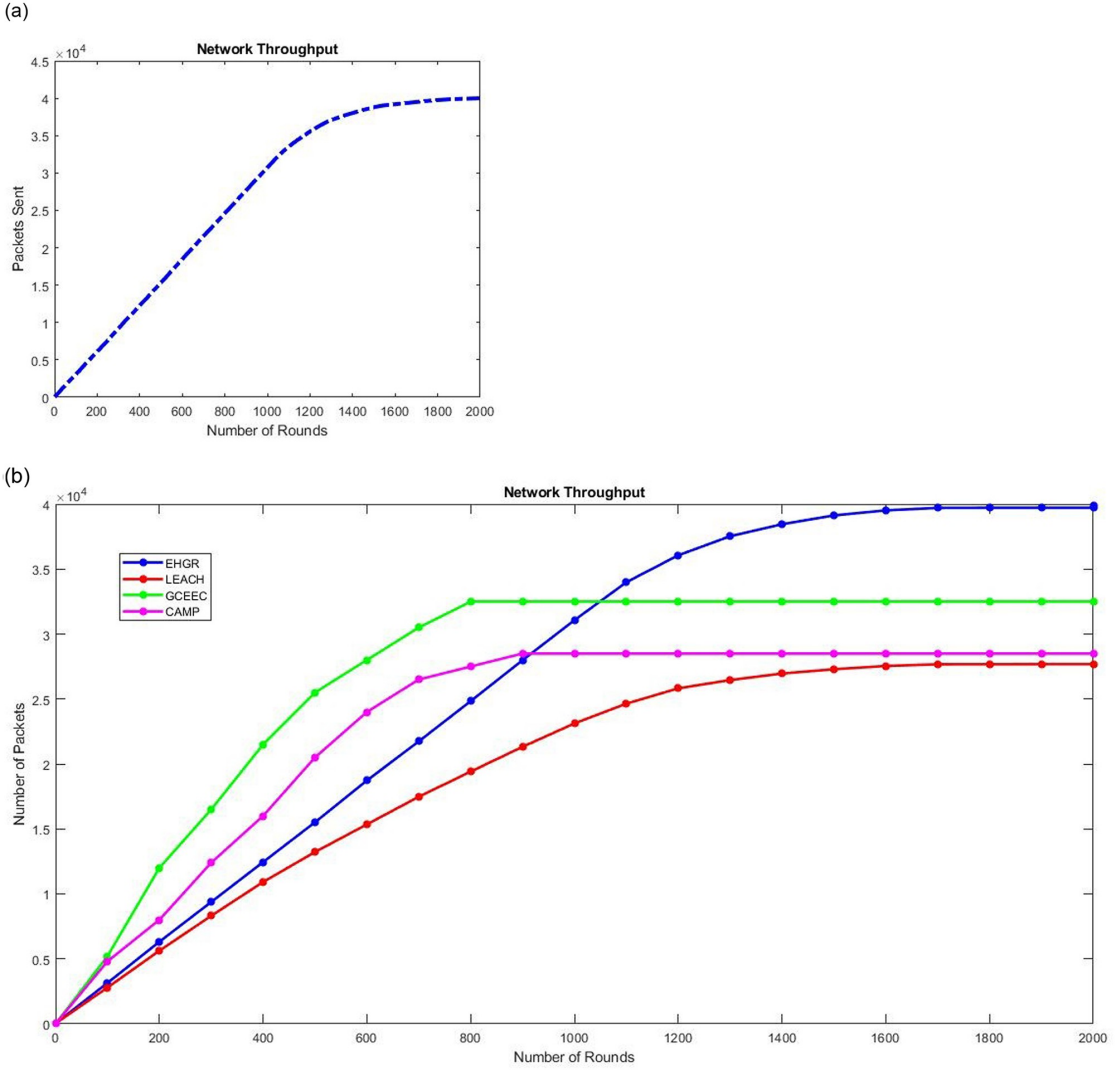
(a). Network Throughput of EHGR: Packets Sent by ECN node to Control Center (b). Comparison of throughput EEHGR with the rest of the routing protocols.

Network throughput is an important indicator for an energy efficient routing protocol and it measure the number packets sent to the control center in different rounds. Examining sub-Fig 7(a) it can be seen that the benefits of offloading computationally intensive tasks to the servicing-uavs results in a substantial increase in th number of packets sent which approximates to 40000 packets per round with a gradual increase. In comparison to the existing routing protocol it can be seen that for both GCEEC and the CAMP first the throughput increases but after 600 rounds the throughput stables to a much lower values in comparison to the throughput of EHGR. Therefore as expected the EHGR outperform the existing routing protocols in generating higher levels of throughput due to its efficient extension of the network coverage by using gateway relay nodes due to which the network coverage is extended and data offloading capabilities which extend network lifetime along with coverage.

#### Network lifetime

**Fig 8:**
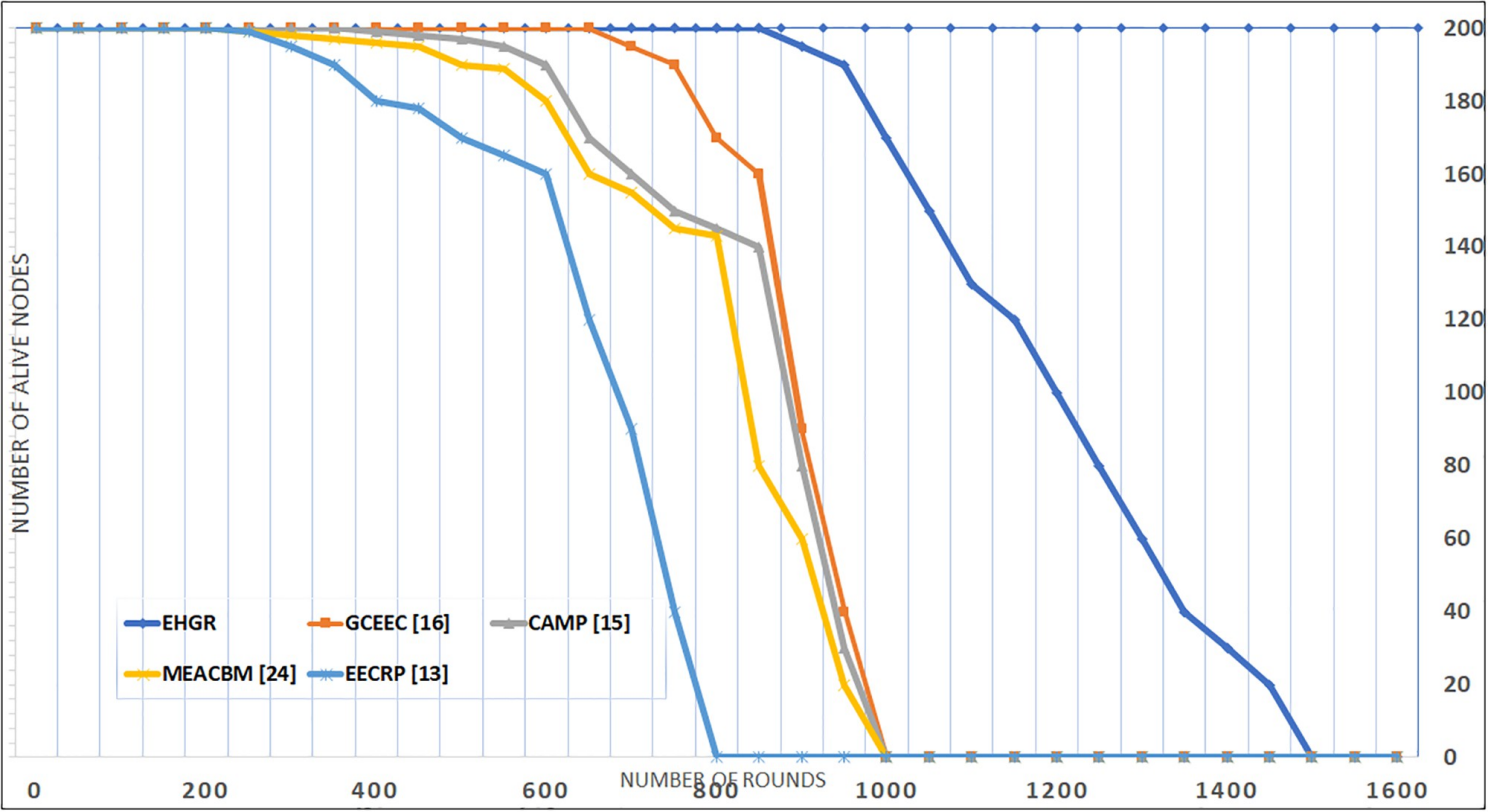
Network lifetime of alive nodes with varying iterations.

The total run time of all the existing approaches presented in this study are mapped against the run time of EHGR in Fig 8. The EHGR has a higher network lifetime against the existing approached this is due to the battery preservation technique adopted. Nodes that indulge in heavy calculations to support network formation stages as mentioned in existing techniques deplete their battery quickly. EHGR however offloads these tasks to the servicing-uavs due to which nodal lifetime is extended. It can be concluded that the uav-assisted wireless sensor networks that perform load balancing in calculations for network formation, zone formations, etc. will enjoy an extended network lifetime as compared to the rest of hierarchical approaches. The algorithm runs for 2000 iterations and the first dead node is reported at the 979th round see Fig 5(a) which is approximately the total life span of the networks established in legacy routing protocols such as LEECH, GCEEC [16], CAMP [8], EECRP [14], MEACBM [17]. Finally the total network lifetime is shown in Fig 7 which shows the benefits of the UAV assisted WSN where the UAV are deployed to extend network lifetime by load balancing.

## Conclusion

In this study a game theory based energy efficient hierarchical gateway routing protocol (EHGR) is presented for ad hoc wireless sensor networks. The study addresses two important challenges for modern uav-assisted wsn’s i.e optimized deployment through game theory matching approach with gateway nodes and second energy efficient routing with load balancing between the servicing unmanned aerial vehicles and the energy centroid nodes. The matching algorithm with the help of gateway nodes achieves an improvement in coverage by ratio of 100% as compared to traditional leach based approaches. The use of energy centroid nodes during data transmission process results in gradual energy dissipation due to which the network lifetime is extended. Due to load balancing of computations during all phases of EHGR between the energy centroid nodes and the unmanned aerial vehicles the decrease in energy dissipation is further controlled and the first dead nodes which is reported at 979th round and last dead node report at 1800th round. The simulation figures presented show a substantial achievement against existing approaches. Overall the EHGR has a remarkable break through at near 1800 rounds for the entire routing process along with energy efficiency such that the total average energy of the entire network remains above 90% for at least 500 rounds. UAV-assisted wsn’s are likely to attract more research in the future. This study will be further extended to explore the behavior of the ad-hoc wsn while using multi hop D2D routing with extended coverage. The routing processes must be continuously improved to address the requirements placed by modern day applications all of which require more computation, traffic prioritization, improved load balancing techniques with extended network lifetime.

## Supporting information

S1 Data (ZIP)
