# Correction: Energy efficient gateway based routing with maximized node coverage in a UAV assisted wireless sensor network

**DOI:** 10.1371/journal.pone.0316593

**Published:** 2024-12-26

**Authors:** Bilal Ahmad, Masroor Ahmed, Nadeem Anjum, Masood Ur Rehman, Naeem Ramzan

The affiliation for the second author is incorrect. Masroor Ahmed is not affiliated with #2 but with #1: Capital University of Science & Technology, Islamabad, Pakistan.
